# Enhanced condensation heat transfer using porous silica inverse opal coatings on copper tubes

**DOI:** 10.1038/s41598-021-90015-x

**Published:** 2021-05-21

**Authors:** Solomon Adera, Lauren Naworski, Alana Davitt, Nikolaj K. Mandsberg, Anna V. Shneidman, Jack Alvarenga, Joanna Aizenberg

**Affiliations:** 1 John A. Paulson School of Engineering and Applied Sciences, Harvard University, Cambridge, Massachusetts 02138 USA; 2 Wyss Institute for Biologically Inspired Engineering, Harvard University, Cambridge, Massachusetts 02138 USA; 3 Department of Mechanical Engineering, University of Michigan, Ann Arbor, Michigan 48109 USA; 4 Department of Chemistry and Chemical Biology, Harvard University, Cambridge, Massachusetts 02138 USA; 5 Department of Health Technology, Technical University of Denmark, 2800 Kongens Lyngby, Denmark

**Keywords:** Engineering, Mechanical engineering

## Abstract

Phase-change condensation is commonplace in nature and industry. Since the 1930s, it is well understood that vapor condenses in filmwise mode on clean metallic surfaces whereas it condenses by forming discrete droplets on surfaces coated with a promoter material. In both filmwise and dropwise modes, the condensate is removed when gravity overcomes pinning forces. In this work, we show rapid condensate transport through cracks that formed due to material shrinkage when a copper tube is coated with silica inverse opal structures. Importantly, the high hydraulic conductivity of the cracks promote axial condensate transport that is beneficial for condensation heat transfer. In our experiments, the cracks improved the heat transfer coefficient from ≈ 12 kW/m^2^ K for laminar filmwise condensation on smooth clean copper tubes to ≈ 80 kW/m^2^ K for inverse opal coated copper tubes; nearly a sevenfold increase from filmwise condensation and identical enhancement with state-of-the-art dropwise condensation. Furthermore, our results show that impregnating the porous structure with oil further improves the heat transfer coefficient by an additional 30% to ≈ 103 kW/m^2^ K. Importantly, compared to the fast-degrading dropwise condensation, the inverse opal coated copper tubes maintained high heat transfer rates when the experiments were repeated > 20 times; each experiment lasting 3–4 h. In addition to the new coating approach, the insights gained from this work present a strategy to minimize oil depletion during condensation from lubricated surfaces.

## Introduction

Vapor-to-liquid phase change condensation is routinely observed in numerous industrial processes^1–11^. For example, in fossil-fueled or coal-fired steam power plants, the low pressure steam that exits the turbine enters a condenser unit and changes its phase to liquid. In a similar fashion, distillation towers sequentially liquefy vapor mixtures into various component liquid hydrocarbons by employing a condenser. The distilled hydrocarbons are later used as energy sources for powering various industries including land, sea, and air transportation. Desalination^12^ is another example that gained traction in recent years due to increased concerns of global water scarcity^13^. The widely used multi-stage flash desalination plants^5^ employ condensers to produce potable water by condensing the vapor that is generated from saltwater (brine). Condensation is equally important in nature. It is observed when plant leaves collect dew in the early morning hours of the summer or when fog forms on windowpanes on humid days. Condensation processes require that the enthalpy of phase change be removed by the working fluid. Compared to other working fluids, water has a significantly higher enthalpy of phase change (2.257 MJ/kg at 373.15 K and 1 atm)^14,15^. Due to its impact in power and process engineering^16–20^, condensation is still an active area of research in spite of its long history that spans over a century.

Phase-change condensation commences when the wall temperature of a surface is decreased below the saturation temperature. In a pure vapor environment where noncondensable gases (NCG) are absent^21–28^ the required subcooling (the degree to which the condenser surface is cooled relative to the surrounding vapor) to initiate condensation is low. NCGs are non-target species such as oxygen and nitrogen gases that do not condense or liquefy at the saturation temperature of water vapor (100 °C at 1 atm). Instead, NCGs, which are driven along with the target species to the liquid–vapor interface due to convection, accumulate near the condensing surface and create a diffusion barrier for phase change condensation^26^. Consequently, when the vapor mixture contains not only the condensing species but also one or more NCGs, the phase change heat transfer coefficient is significantly lower than when the NCGs are absent. In the presence of NCGs, the condensation rate is lower because the condensing species must first diffuse through the concentration boundary layer that coats the vapor side of the interface. The condensing species must first overcome the mass transfer resistance posed by the concentration boundary layer created by accumulation of gases near the liquid–vapor interface^29^.

**Figure 1:**
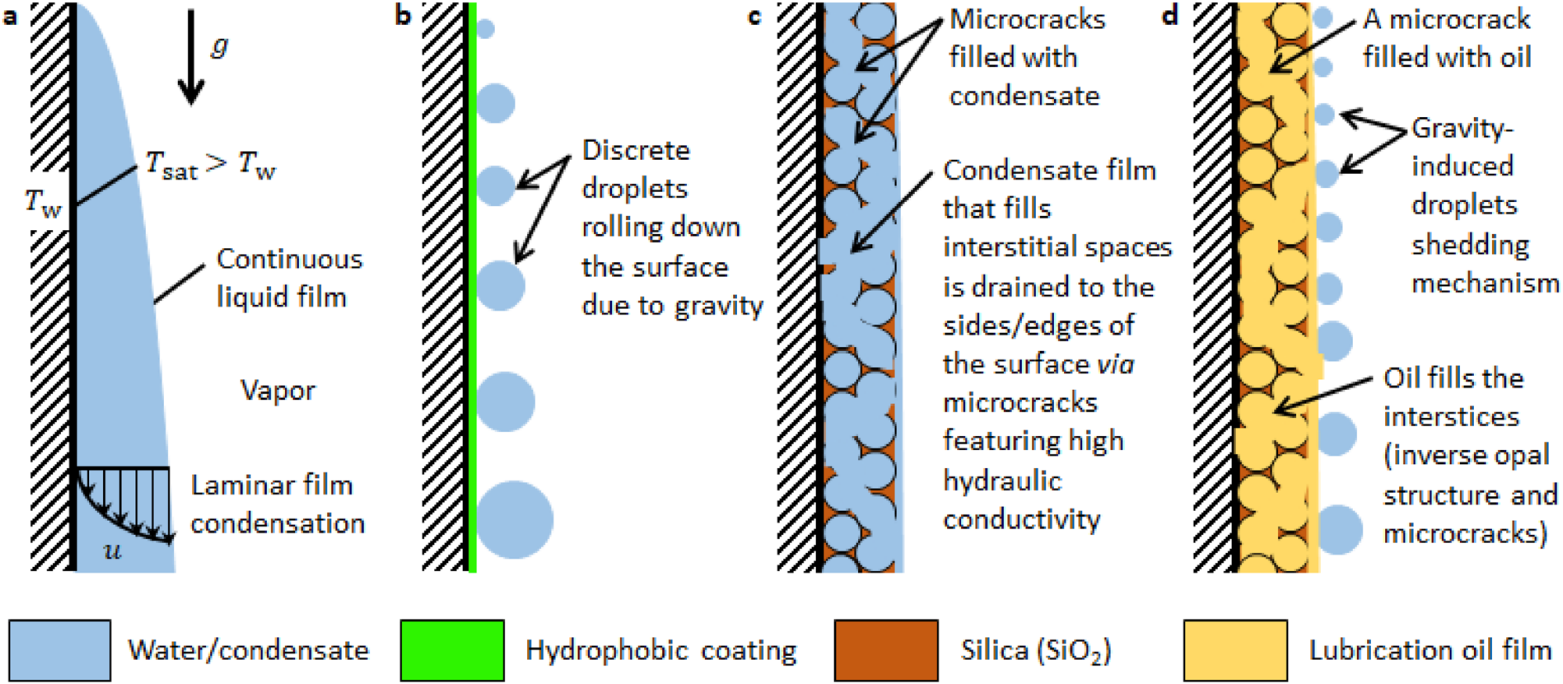
Schematic depictions of different modes of condensation. (**a**) Filmwise condensation (FWC) where the condensate forms a continuous liquid film on a chemically clean (high surface energy) surface. The wall is maintained at a lower temperature ($T_{{\text{w}}}$) than the surrounding saturated vapor ($T_{{{\text{sat}}}}$). The temperature and velocity profiles are shown for laminar film condensation where the flow-rate based Reynolds number is < 30. (**b**) Dropwise condensation (DWC) on hydrophobic (low surface energy) surface. It is essential for dropwise condensation that some promoter material, for example organic compound, be present on the surface. Periodic removal of large droplets clears the surface for renewed droplet nucleation and growth. (**c**) Inverse opal condensation (IOC) on a porous inverse opal structure. Condensate seeps into the porous structure by displacing the air in the pores. Preferential condensate transport through high hydraulic conductivity micro cracks improves the heat transfer rate. (**d**) Condensation on a slippery liquid-infused porous surface (SLIPS). The porous interstices are impregnated with a chemically matched oil. Compared to DWC, droplets depart with smaller radius and higher frequency in SLIPS condensation.

There are two clearly distinguishable ideal modes of condensation of water vapor on cold metallic surfaces, namely, filmwise condensation (FWC)-when the condensed vapor is distributed as a continuous liquid film with characteristic thickness ≈ 100 μm (Fig. 1a), and dropwise condensation (DWC)-when the condensate liquid is in the form of discrete droplets (Fig. 1b). A series of publications in 1934 and 1935 by Nagle et al.^30–33^ established that pure steam condensing on a chemically clean surface always forms a continuous film and that the presence of impurities which render the surface nonwetting is essential for the formation of discrete droplets. Since then, it is well understood that FWC occurs on high surface energy metallic surfaces and their oxides^34–36^. Whereas DWC occurs when the surface is coated with a promoter material (for example, chemical treatment with a hydrophobic silane or fluorocarbon)^37–40^. In the design of condensers, the function of which is to cool a vapor stream in order to convert it to liquid phase, there is a great incentive to promote the breakup of the condensate film into discrete droplets. Numerous studies have demonstrated that phase-change heat transfer during DWC is nearly an order of magnitude higher than FWC in a pure vapor environment since in the latter mode of condensation, the continuous liquid film increases the overall thermal resistance by adding a conduction resistance. In both FWC and DWC, condensate liquid is drained vertically when gravity overcomes pinning forces. For DWC, this occurs when the drop radius becomes comparable to the capillary length of water (≈ 2.7 mm at 25 °C and 1 atm). The periodic surface clearing and sweeping action performed by the large droplets renews finite-size regions of the surface for restart of the time-dependent condensation process, thereby reactivating nucleation sites to improve the overall condensation heat transfer.

Unfortunately, the promoter materials or hydrophobic coatings that are commonly used for DWC have proven to have finite lifetime. They wear off rather quickly due to viscous drag or friction exerted on the coating by the sweeping action of the droplet. Depending on the working condition inside the condenser, the performance benefits of hydrophobic coatings could last only few hours^37,41^. Especially under harsh industrial working conditions (for example, pressurized superheated steam), promoters can breakdown with minor impurities in the steam or due to erosion by water droplets. For example, Hampson and Ozisiks^41^ showed that a brass surface treated with oleic acid exhibits dropwise condensation of saturated steam for only ≈ 3 h. Industrial condenser surfaces, however, are required to last longer than what has been demonstrated thus far using surface treatment techniques. Much research has been devoted in understanding the time interval during which promoter coatings remain effective. The manner of their breakdown in industrial conditions has since been undertaken with the aim to produce long-lived or near permanent dropwise condensation. Thick hydrophobic coatings can mitigate the problem of wear-and-tear. But simply increasing coating thickness is generally not favorable since such an approach increases the overall conduction resistance. Prior studies have shown that due to the increase in conduction resistance, hydrophobic coatings in excess of 20 μm tend to offset the heat transfer augmentation effect obtained from DWC. Despite numerous studies since it was first reported in the 1930s^42^, creating durable hydrophobic coatings for DWC is still a challenge that is not resolved to date.

In this work, we demonstrate a different approach that does not require a chemical functionalization with a hydrophobic coating. We create a porous silica structure known as an inverse opal to allow vapor to condense in its energy favorable state as shown in Fig. 1c. The condensate liquid that instantaneously spreads and seeps into the porous structure is transported away from the surface through micro cracks that arise naturally during the fabrication process. Due to the hydrophilic nature of the substrate and inner pore surfaces, these cracks have high hydraulic conductivity (low flow resistance) that favors rapid preferential condensate transport in the axial direction that is beneficial for phase change. Hereinafter, we refer this mode of condensation as inverse opal condensation (IOC). This material design provides a durable solution by eliminating the need for a promoter coating. The concept of actively controlling the thickness of the condensate film using porous structures has been reported in previous studies^43–48^. The current work validates the concept by conducting heat transfer measurements, through which we show that the porous inverse opal coatings on copper tubes show comparable heat transfer rates to state-of-the-art hydrophobic-coated surfaces for DWC, a sevenfold increase compared to surfaces exhibiting FWC. Furthermore, we chemically functionalized and impregnated the porous structure with a lubrication film to create slippery liquid-infused porous surfaces (SLIPS) for condensation, shown schematically in Fig. 1d. Droplet shedding was facilitated significantly (i.e., faster droplet shedding at smaller length scale and higher frequency compared to those in DWC). Due to improved droplet mobility, the vapor-side heat transfer rate was improved further in SLIPS condensation compared to both DWC and IOC. The insights gained from this work present an opportunity to solve one of the long-standing durability concerns of modern condenser surface coatings.

### Sample fabrication

**Figure 2:**
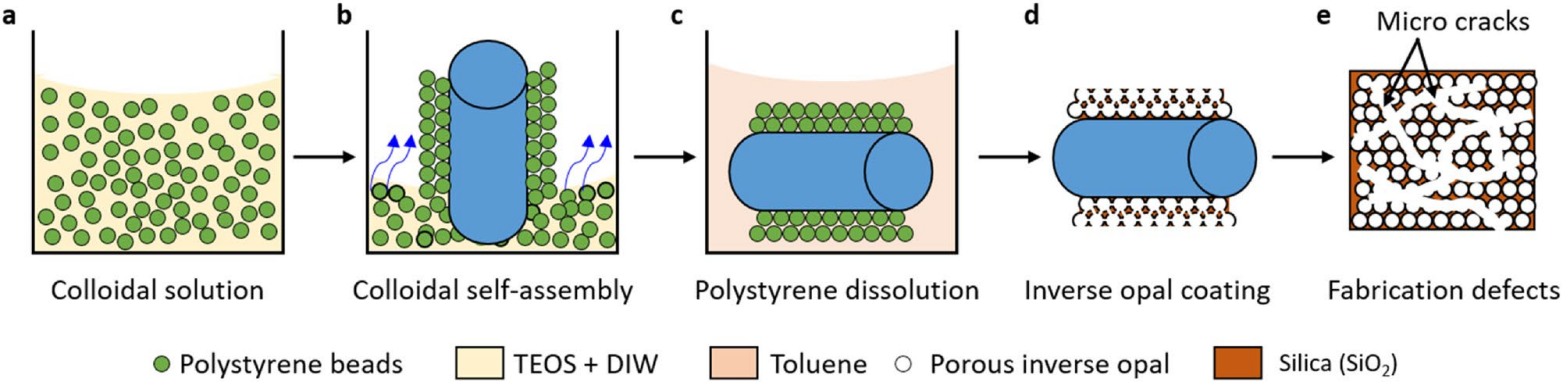
Schematic depiction of the fabrication procedure to produce inverse-opal-coated copper tubes. (**a**) Colloidal solution is prepared by adding 0.5 wt% colloidal polystyrene particles (395 nm diameter) into a solution of 99.4 wt% deionized water and 0.1 wt% tetraethyl orthosilicate (TEOS). (**b**) A copper tube coated with silica via atomic layer deposition (ALD) and oxygen plasma cleaned is inserted vertically in the colloidal solution to allow colloidal self-assembly as the solution evaporates. (**c**) The polystyrene beads are dissolved by immersing the copper tube in toluene. (**d**) A porous inverse opal structure composed of a network of voids is created when the sacrificial polystyrene beads dissolve in toluene. (**e**) During the drying stage, interconnected micro cracks featuring large hydraulic conductivity form between islands of inverse opals.

Using a bottom-up colloidal co-assembly technique^49–51^, we coated a copper tube (length = 60 mm, external diameter = 6.35 mm, internal diameter = 4.57 mm) with inverse opal structures, a highly porous material coating consisting of sub-micron voids in a support matrix, here silica. In brief, the copper tube is wet-cleaned using solvents (acetone, ethanol, isopropanol, and deionized water sequentially) and a thin (≈ 4 nm) layer of silica is deposited on the surface using atomic layer deposition (ALD). Additional wet-cleaning and oxygen plasma cleaning are performed in order to facilitate wetting of the colloidal solution and subsequent self-assembly on the copper surface. The clean silica-coated copper tube is immersed in a colloidal solution consisting of templating polystyrene beads together with tetraethyl orthosilicate (TEOS), the precursor for the background matrix, as shown in Fig. 2a. The colloids self-assemble into a face-centered cubic lattice (fcc) as the solution evaporates (Fig. 2b) with hydrolyzed TEOS forming a silica network in the interstitials between the assembled spheres. The polystyrene beads are then dissolved in toluene (Fig. 2c) resulting in a porous silica coating (Fig. 2d). During the drying stage, fabrication defects arise in the form of interconnected micro cracks between islands of inverse opals as shown in top-down schematic view of the porous inverse opal structure (Fig. 2e). See “Materials and methods” for more details regarding material synthesis and fabrication.

**Figure 3:**
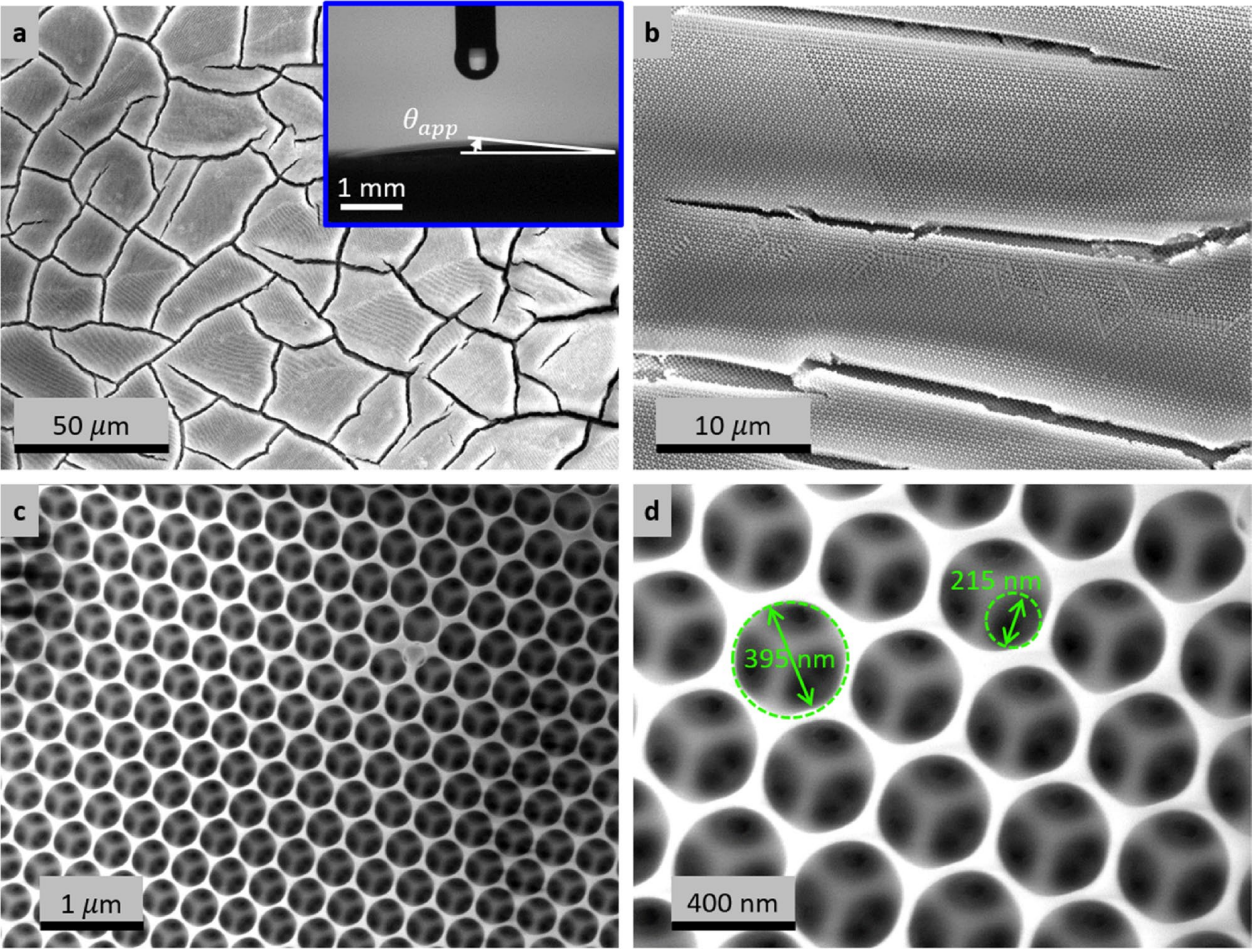
Scanning electron microscope (SEM) images of an inverse opal coating at different magnifications. The low magnification SEM images in (**a**) and (**b**) show fabrication defects (cracks) that formed when the supporting matrix shrank during drying. The inset in (**a**) shows a vanishing contact angle for a water droplet deposited on the porous structure. The high magnification images in (**c**) and (**d**) show the pores which resulted from dissolution of the sacrificial polystyrene beads (≈ 395 nm diameter). The contact points between the polystyrene beads that was not wetted by the solution during co-assembly created interconnected pores (≈ 215 nm diameter).

Typical scanning electron micrograph (SEM) images of the porous silica inverse opal coating at different magnifications are presented in Fig. 3a–d. During co-assembly of the polystyrene beads (≈ 395 nm diameter), the contact area that remained dry (i.e., without being wetted by the solution) resulted in interconnected network of holes/voids (≈ 215 nm diameter) as shown in Fig. 3d. Since silicon dioxide is intrinsically hydrophilic^52^, the resulting inverse opal coating is superhydrophilic and a millimeter size water droplet spreads with vanishing apparent contact angle ($\theta_{app}$) as shown by the inset in Fig. 3a. Material shrinkage-induced cracks formed between islands of inverse opals (Fig. 3a,b). Surface wettability studies (Fig. S1) and additional SEM images including post condensation SEM characterization (Fig. S2) are provided in Supporting Material Section S1.

Based on the SEM measurements, the typical thickness of the inverse opal coating is ≈ 8–12 μm.. Looking at the fluidic transport mechanism, small coating thickness gives rise to larger flow/viscous resistance. Inversely, increasing the coating thickness increases the conduction resistance for heat transfer. Consequently, both of these effects (i.e., coupled momentum and energy equations) need to be considered to optimize coating thickness for condensation, which can be achieved in the assembly process. For example, the size of the beaker containing the copper tube can be increased to reduce the influence of the beaker walls and thus reduce the thickness. To increase the thickness, multiple colloidal assembly steps can be performed in sequence.

## Results and discussion

**Figure 4:**
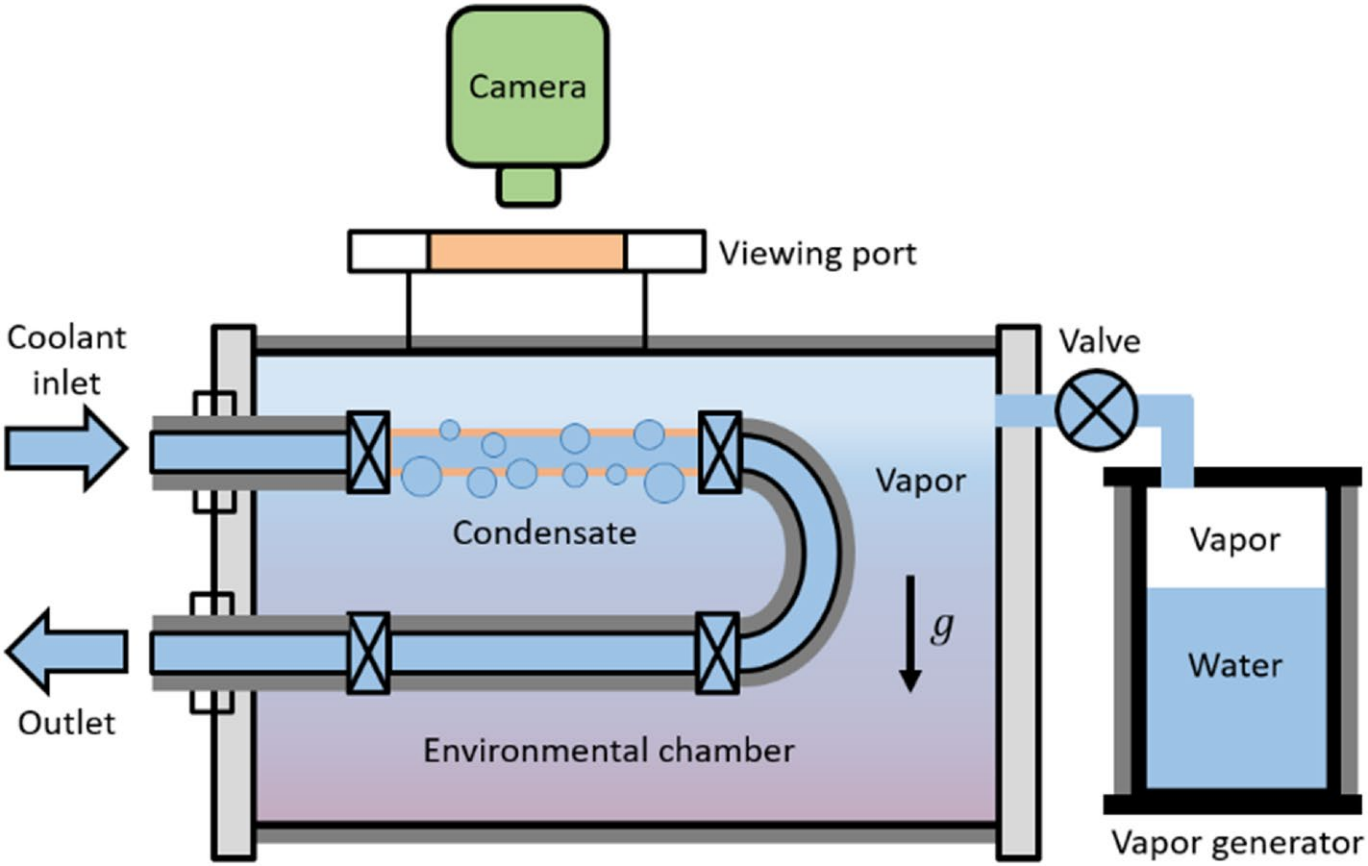
Schematic of the experimental setup constructed for phase change condensation study. The environmental chamber maintains saturation condition by eliminating NCGs from the system. The vapor that enters the chamber condenses on the copper tube that is maintained below the saturation temperature by circulating chilled water from a water bath heat exchanger. To minimize heat loss to the surrounding environment, both the chamber and the vapor generator are insulated using a fiberglass sheet. The chamber has a view port to observe droplet nucleation, growth, and departure.

We built an experimental setup to measure the heat transfer rate and visualize droplet nucleation, growth, and departure. The main components are an environmental chamber, vapor generator, and cooling system (Fig. 4). In addition to maintaining the chamber at saturated conditions (saturated temperature at the corresponding pressure), the custom-made stainless steel environmental chamber is designed to eliminate non-condensing gases from the system. The chamber is externally insulated using low thermal conductivity fiberglass sheet (9333K71, McMaster-Carr) to reduce heat loss to the surrounding environment. During the experiment, the chamber walls were heated slightly above the saturation temperature by turning on a rope heater that is wrapped around the chamber under the insulation. This was necessary to prevent droplet condensation on the internal walls of the chamber which can potentially interfere with visualization. Images of droplet nucleation, growth, and departure were captured at 0.2 frames per second (fps) using a DSLR camera. Details of the environmental chamber and associated instrumentation is discussed in Supporting Material Section S2, Fig. S3. Additionally, typical images showing the different modes of condensation is given in Fig. S4 (Supporting Material Section S2).

In preparation for the experiment, the water in the vapor generator was degassed via vigorous boiling. The degassed water in the vapor generator was maintained at saturated conditions at ≈ 85 °C and ≈ 58 kPa. After attaching the coated copper tube to the cooling water circulation line, the entire system was evacuated using a roughing pump to remove NCGs. The absence of NCGs was confirmed by matching the chamber saturation temperature with the corresponding saturation pressure. During the experiment, hot steam (vapor) was allowed to enter the chamber from the vapor generator by slowly opening a needle valve. Vapor inlet was controlled to allow saturation conditions inside the chamber. The copper tube was maintained below the chamber saturation temperature by circulating chilled water whose inlet and outlet temperatures were measured using calibrated thermocouples as shown in Fig. S5 (Supporting Material Section S3). For reliable heat transfer measurements, a 0.2–2.5 °C temperature difference was maintained between incoming and outgoing cooling water by controlling the flow rate of the coolant. In addition to the temperature of the working fluid, the wet-bulb and dry-bulb temperatures of the chamber, flow rate, and chamber pressure were measured and recorded using a data acquisition system.

**Figure 5:**
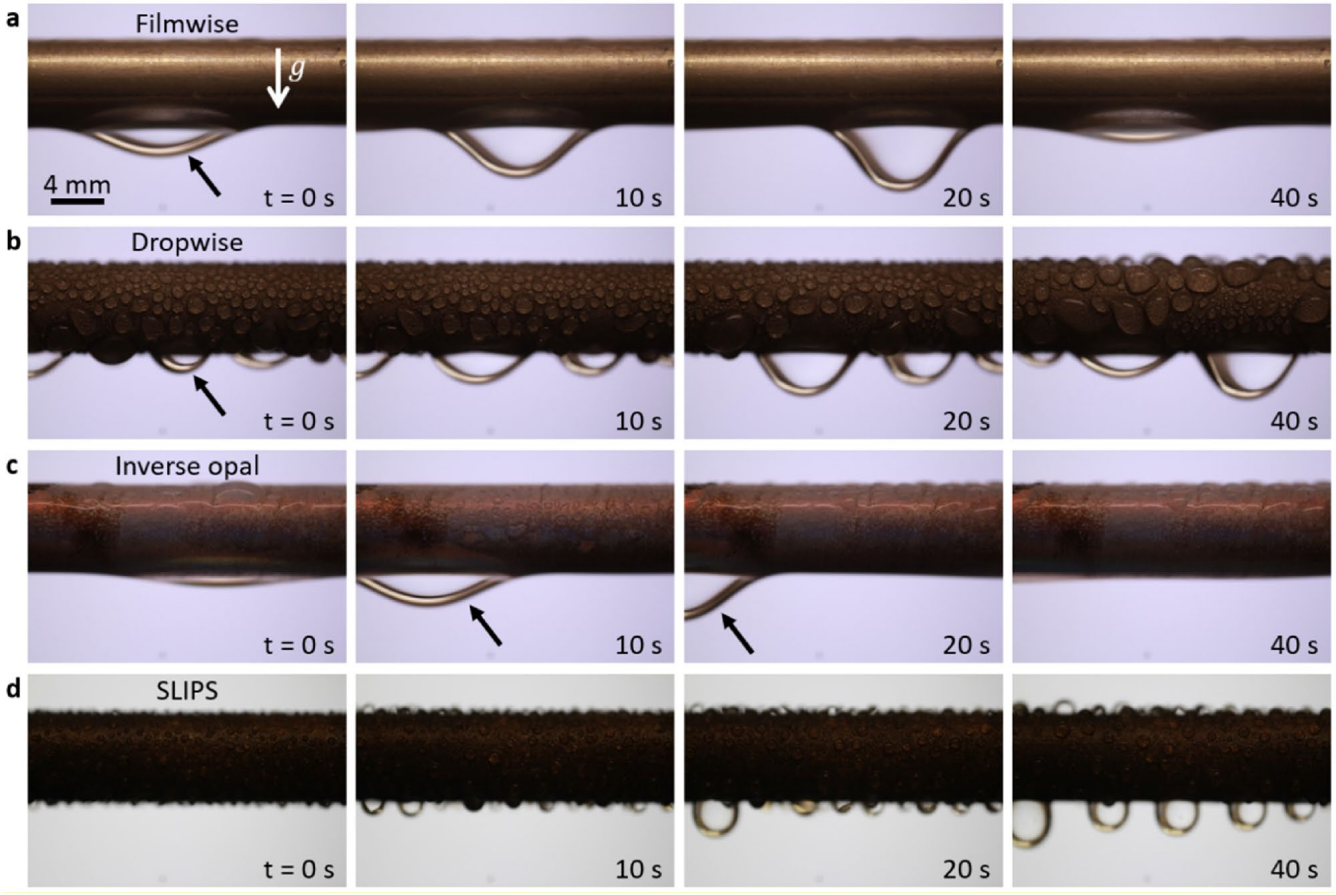
Time-lapse images of water condensation on copper tubes subject to varying surface treatments, showing different modes of condensation. (**a**) FWC on a smooth plasma treated copper tube, (**b**) DWC on smooth hydrophobized copper tube, (**c**) IOC on silica inverse opal-coated copper tube, and (**d**) SLIPS condensation on oil-impregnated porous structure. In all cases except IOC, condensate was drained vertically downwards when gravity overcame pinning forces. During IOC, however, the condensate was transported preferentially in the axial direction through the cracks. Depending on the geometry, size, and orientation of the crack, the condensate moved axially to the right or to the left, but never vertically.

Different modes of condensation ensue depending on the type of the surface coating (Fig. 5). When the smooth copper tube was oxygen plasma treated, steam condensed by forming a continuous liquid film (Fig. 5a). The condensate film shown by the arrow at t = 0 s grew large and sheds due to gravity at t = 20 s. When the smooth copper tube was hydrophobized via silane treatment (trichloro(1H,1H,2H,2H-perfluorooctyl)silane, Sigma Aldrich), vapor condensed by forming discrete water droplets (arrow, t = 0 s, Fig. 5b). Condensate droplets detached when their radius reached the capillary length (≈ 2.7 mm for water). In both the filmwise mode (Fig. 5a) and dropwise mode (Fig. 5b), condensate was drained vertically due to gravity. Additional images are available in Fig. S4 (Supporting Material).

When the copper tube was coated with porous silica inverse opals, the condensate seeped into the interconnected pores since both the underlying substrate (silica-coated copper) and silica inverse opal matrix are inherently hydrophilic. Importantly, the condensate was transported rapidly in the axial direction through the interconnected cracks to the ends of the tube. The droplet shown by the arrow in Fig. 5c (t = 10 s) moved to the left (t = 20 s) even though we observed condensate transport to the right or to the left depending on the dimension, geometry and orientation of the cracks. We attribute the axial transport to the high hydraulic conductivity of the cracks when compared to the interstitial pores. This condensate transport mechanism is distinctively different from FWC (Fig. 5a) and DWC (Fig. 5b) wherein the condensate film is drained vertically due to gravity.

The droplet shedding mechanism reported recently by Anderson et al.^53^ is also different from our observation. Here the coating has homogeneous wettability (due to the superhydrophilic character of the inverse opal coating) whereas Anderson et al.^53^ used biphilic surfaces (hydrophilic base with hydrophobic tips) to connect nucleating droplets via liquid bridges. When the inverse opal structure was chemically functionalized with silane and impregnated with silicone oil (100 cSt at 25 °C, Sigma Aldrich), vapor condensed by forming highly mobile water droplets that depart at smaller length scale (Fig. 5d). Compared to the classical DWC, prior studies have shown that SLIPS condensation improves the heat transfer rate by reducing the departure radius by nearly 50%^54,55^. In addition to enhancing condensation, the dynamic liquid–liquid interface^56^ in SLIPS has been demonstrated for other applications, for example, water harvesting^57,58^, ice adhesion^59,60^, and biofouling^61,62^.

**Figure 6:**
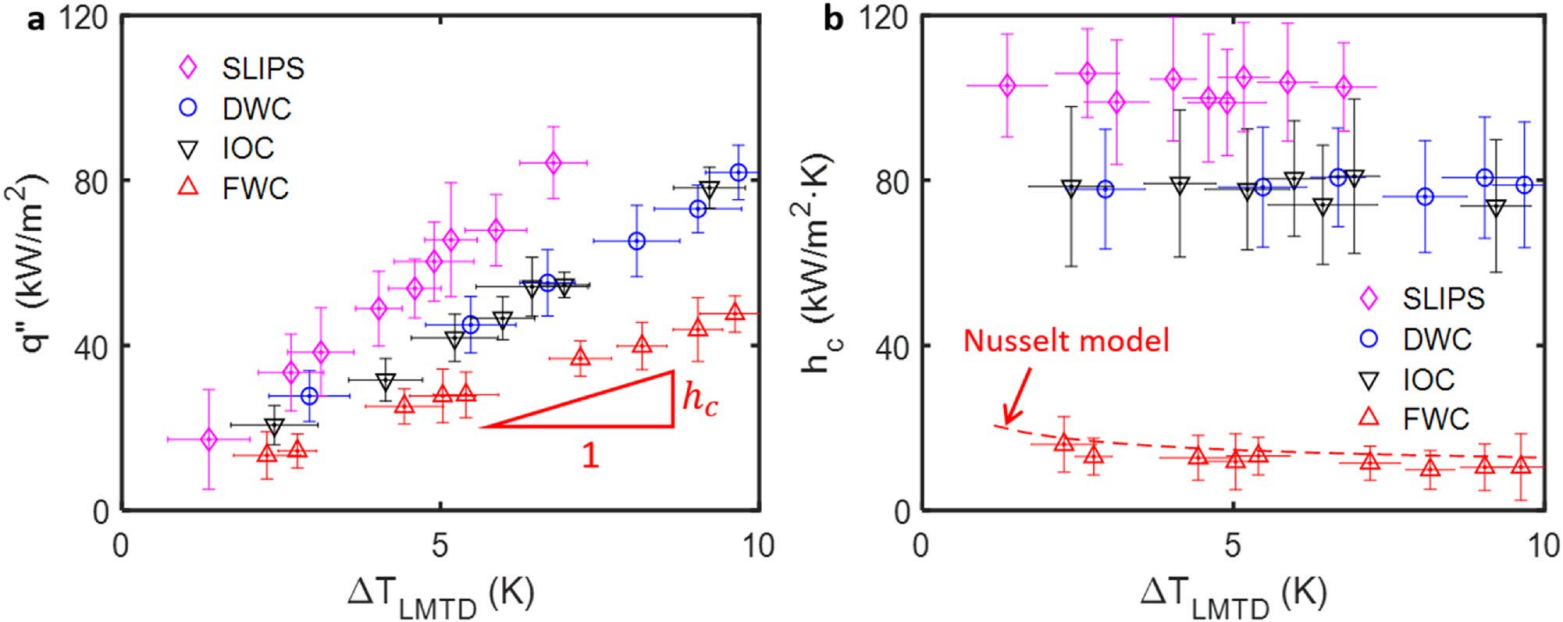
Heat transfer measurement. (**a**) Heat flux and (**b**) heat transfer coefficient as a function of logarithmic mean temperature difference. The slope in (**a**) is the heat transfer coefficient ($h_{c}$). The heat transfer coefficient for DWC and IOC are nearly identical at ≈ 80 kW/m^2^ K; a sevenfold increase from FWC (≈ 12 kW/m^2^ K). The heat transfer coefficient increased further by ≈ 30% to ≈ 103 kW/m^2^ K when the silica pores were impregnated with oil (SLIPS condensation). The classical Nusselt model for laminar film condensation is used to validate our heat transfer measurements.

Since our experiments were neither constant temperature (isothermal) nor constant heat flux (isoflux), we used logarithmic mean temperature difference as a measure for the average temperature difference driving the phase-change process. For the inlet ($T_{1}$) and outlet ($T_{2}$) coolant temperatures (Fig. S6, Supporting Material) and chamber temperature ($T_{{{\text{sat}}}}$), the logarithmic mean temperature difference is defined as $\Delta T_{{{\text{LMTD}}}} = \left( {T_{2} - T_{1} } \right)/\ln \left( {\Delta T_{2} /\Delta T_{1} } \right)$ where $\Delta T_{2} = T_{2} - T_{{{\text{sat}}}}$ and $\Delta T_{1} = T_{1} - T_{{{\text{sat}}}}$. The heat rejected by the steam per unit area or heat flux ($q^{\prime\prime}$) was calculated from internal flow analysis. The heat released by the vapor during condensation (Fig. S7, Supporting Material) was carried away by the chilled water. All data were acquired at steady-state conditions where all temperature fluctuations (wet-bulb, dry-bulb, inlet and outlet) and chamber pressure fluctuations were ≤ 0.25 °C/min and ≤ 0.1 kPa/min, respectively. The acquired steady-state data was time-averaged to yield a single data point shown in Fig. 6 (see Supporting Material Section S4 for details regarding data reduction).

The heat flux as a function of logarithmic mean temperature for the different modes of condensation is shown in Fig. 6a. The smooth copper tube was plasma cleaned for FWC, whereas it was silane treated for DWC. In our experiments, both DWC and IOC demonstrate higher heat transfer coefficient than FWC. Among the different modes of condensation, SLIPS condensation performed the best since it removed high heat fluxes at relatively smaller logarithmic mean temperature difference (Fig. 6a). The slope of heat flux versus logarithmic mean temperature difference is the heat transfer coefficient ($h_{c}$), which measures the rate at which heat is removed per unit area per unit temperature difference or subcooling^15^. The heat transfer coefficient as a function of the logarithmic mean temperature difference for the different modes of condensation is shown in Fig. 6b. In our measurements, the heat transfer coefficient for DWC and IOC were nearly identical (within measurement uncertainty) at 79 ± 14 kW/m^2^ K and 78 ± 17 kW/m^2^ K, respectively. This is nearly a sevenfold increase from FWC (12 ± 6 kW/m^2^ K) and agrees with prior models for DWC^17,18,63^. We attribute the high heat transfer rates in IOC to preferential condensate transport in the axial direction featuring high hydraulic conductivity cracks. When the porous inverse opal coating was functionalized and impregnated with a lubrication film, the phase-change heat transfer coefficient increased by an additional 30% to 103 ± 13 kW/m^2^ K. This enhancement in heat transfer rate agrees with prior studies^64^. We attribute the improvements in heat transfer rate in SLIPS to smaller departure radius and higher departure frequency of falling droplets when compared to DWC. The solid–liquid composite material design in SLIPS improved droplet mobility (≈ 1–2° contact angle hysteresis) by providing an atomically smooth and chemically homogeneous liquid–liquid interface^65,66^. We validated our measurements by comparing the heat transfer coefficient for FWC on a smooth oxygen plasma treated copper tube with the classical Nusselt model (Supporting Material Section S5, Fig. S8) for laminar thin film condensation (red dashed line, Fig. 6b)^36^. The error bars in Fig. 6 are calculated through error propagation analysis by combining systematic and random errors for one standard deviation (Table S1, Supporting Material Section S6).

**Figure 7:**
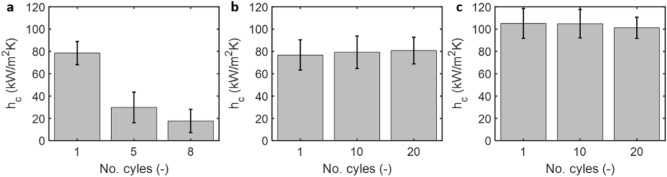
Coating durability. (**a**) The silane coating on the copper tube degraded after 5–8 experimental runs. The heat transfer coefficient for DWC decreased from ≈ 79 kW/m^2^ K (first experiment) to ≈ 13 kW/m^2^ K (eighth experiment). The enhanced heat transfer coefficient for the inverse opal coated copper tube (**b**) and the oil-impregnated copper tube (**c**) remained nearly the same at ≈ 80 kW/m^2^ K (IOC) and ≈ 102 kW/m^2^ K (SLIPS) when condensation experiments were conducted for the first, tenth, and twentieth times. This result shows the durability of our coating.

We tested the durability of the coating by repeating the experiments for > 20 times with each condensation experiment lasting 3–4 h. The performance benefits of the smooth hydrophobic silane coating on the copper tube did not last long. Initially, the smooth hydrophobized (silane treated) copper tube exhibited dropwise condensation. However, the condensation resorted to a mixed mode (filmwise and dropwise) after 5–8 experimental runs likely due to the deterioration of the hydrophobic coating. In our measurements, the heat transfer coefficient for the first, fifth, and eighth experiments were 79 kW/m^2^ K, 30 kW/m^2^ K and 13 kW/m^2^ K (Fig. 7a). The performance enhancement from inverse opal coating with or without oil impregnation lasted significantly longer (Fig. 7b,c). The heat transfer coefficient for IOC (≈ 80 kW/m^2^ K, Fig. 7b) and SLIPS condensation (≈ 102 kW/m^2^ K, Fig. 7c) remained nearly constant when the experiments were repeated for the tenth and twentieth times. In addition to the heat transfer measurements, we characterized the tube surface post-condensation using SEM (Supporting Material Section S1). We did not observe noticeable topographical changes on the inverse opal coating; suggesting its durability.

In addition to heat transfer measurements, we visualized phase-change condensation using an environmental scanning electron microscope (ESEM, EVO 50 ESEM, Carl Zeiss Microscopy GmbH). For this *in-situ* characterization, the ESEM was maintained at saturated conditions at 7.4 °C and 1015 Pa. After securely attaching the test sample to a water-cooled Peltier stage (Deben TM-1000 Coolstage, Deben UK Limited) using a vacuum-compatible carbon tape, the chamber was evacuated to remove NCGs. The cold stage was maintained 2 °C below the chamber saturation temperature. Backscatter detection mode (CZ-BSD) with high gain and a 25 kV beam potential was used for visualization/imaging. The probe current was maintained at ≤ 2.0 nA to suppress evaporation induced by the beam power. To improve visualization, a 500 μm fixed pressure lower aperture was aligned in series with a 1000 μm variable pressure upper aperture. Using the built-in camera, images of droplet nucleation and growth were captured at 10 fps.

**Figure 8:**
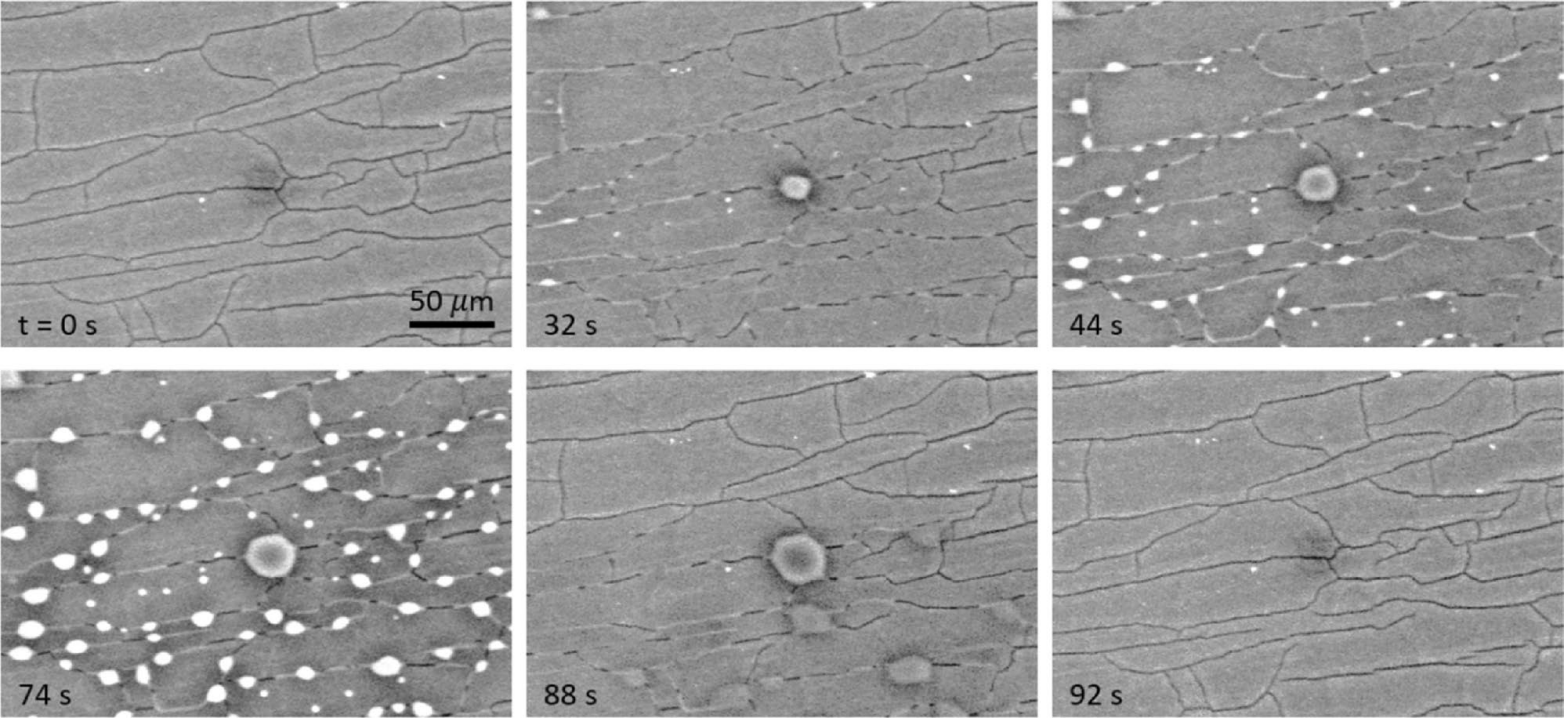
Environmental SEM. The test sample was attached to a cold stage that was subcooled by 2 °C. Time-lapse images show that vapor condensed preferentially near cracks (t = 32 s and 44 s). When condensation continued, the condensate overflowed (t = 74 s) the porous structure. The condensate was transported through the cracks (t = 88 s) and the surface dried out (t = 92 s) when vapor supply was discontinued.

The time-lapse images in Fig. 8 show that droplet nucleation starts preferentially near fabrication defects. The cracks which were empty at t = 0 started to fill with condensate water at t = 32 s. As condensation continued, the condensate liquid started to overflow from the cracks as shown in the time-lapse images at t = 44 s and t = 74 s. When vapor supply was discontinued, the condensate that filled the cracks receded into the interconnected pores (t = 88 s). Eventually, the surface dried out at t = 92 s when the condensate seeped into the porous structure and drained through the cracks or evaporate from the surface. Faster condensate removal through the cracks refreshed the surface and prepared it for renewed droplet nucleation and growth. These results suggest that hierarchical (two-level) porosity, which can be achieved by engineering cracks in colloidal crystals^51^, has a potential for faster condensate removal that can translate to improved phase-change condensation heat transfer rate.

## Conclusions

In summary, we coated copper tubes with silica inverse opals (≈ 8–12 μm thickness) and characterized the phase-change heat transfer rate. This approach does not require a hydrophobic coating; instead it takes advantage of the hydrophilicity of the porous structure to allow vapor to condense in its energy favorable state. Importantly, our material design facilitates condensate removal by enabling preferential condensate transport in the axial direction through fabrication defects which were created fortuitously due to material contraction during the drying stage of the fabrication protocol. Experimentally, we observed condensate transport axially along the copper tube. We attribute the enhanced condensate transport to the large hydraulic conductivity of the cracks when compared to the pores in the inverse opal structure. This preferential axial condensate transport is distinct from typical FWC and DWC where condensate is drained vertically due to gravity. After validating our heat transfer measurements with the classical Nusselt model for laminar thin film condensation, condensation experiments on inverse opal coated copper tubes were conducted. The heat transfer coefficient for inverse opal condensation was ≈ 80 kW/m^2^ K; a sevenfold increase from filmwise condensation (≈ 12 kW/m^2^ K). This heat transfer rate is nearly the same to state-of-the-art DWC. We attribute the high condensation heat transfer rates in inverse opal coated copper tubes to the preferential condensate transport in the axial direction. Importantly, when condensation experiments were repeated > 20 times with each experimental run lasting 3–4 h, the inverse opal coated tubes maintained high heat transfer rate (≈ 80 kW/m^2^ K), indicating durable material design due to the absence of the degradable hydrophobic coating necessary for DWC. In-situ ESEM visualization shows that condensate droplets that form preferentially near fabrication defects are transported away from the condensing surface through micro cracks featuring high hydraulic conductivity. When the pores were chemically functionalized and impregnated with a lubrication film, the heat transfer coefficient increased further by an additional 30% to 103 kW/m^2^ K. In practice, which surface to use (IOC vs SLIPS) will depend on the constraints of the application, for example whether lubricant is compatible with the application and if replenishment of the lubricant is feasible, as it naturally depletes over time. Miscibility of the lubrication film with the condensate is another concern that has to be taken into consideration when selecting the appropriate mode of condensation for a particular application. The insights gained from this work can be used to guide material design for durable condenser surfaces for industrial application. It will be interesting to understand how the crack size and shape affect the discussed properties in order to optimize the vapor-side condensation heat transfer coefficient. The pore dimension may also be a critical length scale that requires optimization. Further testing of the longevity and mechanical properties of the coating will be essential for industrial applications, depending on the actual temperature and pressure the condensing surface may be exposed to.

## Materials and methods

### Colloidal suspension

Colloidal suspension composed of 0.5 wt% colloids (395 nm diameter, –COOH terminated polystyrene spheres), 0.1 wt% hydrolyzed tetraethoxy silane solution (TEOS, Si(OC_2_H_5_)_4_) and 99.4 wt% de-ionized water (DIW) was prepared. The hydrolyzed TEOS solution was prepared by mixing TEOS, ethanol, and 0.1 M hydrochloric acid (HCl) at 1:1:1 ratio. The solution was thoroughly mixed by stirring it for 24 h using a magnetic stirrer at 550 rev/min (rpm). Finally, the solution was added to the colloidal suspension before inserting the 15 min plasma treated copper tubes inside the solution for colloidal co-assembly.

### Silica inverse opals

Copper tubes (length = 60 mm, external diameter = 6.35 mm, internal diameter = 4.57 mm) were sonicated (Branson M8800, Thermo Fisher Scientific) in an acetone bath for 15 min. This was followed by sequential cleaning using acetone, ethanol, isopropanol (IPA), DIW and blow-drying with compressed air. Next, the copper tubes were coated with ≈ 4 nm silicon dioxide (SiO_2_) via ALD. During ALD, the tubes were lifted slightly on one end by a metal wire to ensure uniform coverage. After ALD, the copper tubes were oxygen plasma treated (PE-200, Plasma Etch) for 15 min and immersed in a 12 ml glass vial filled with colloidal suspension. The assembly was placed in a constant temperature (65 °C) convection oven on a vibration free table. After allowing the solution to evaporate fully for > 3 days, the colloidal coated copper tubes were taken out of the oven and immersed in toluene (C_6_H_5_CH_3_, Sigma Aldrich) for > 24 h to dissolve the sacrificial polystyrene beads. Typical thickness of the porous silica inverse opal coating deposited on the copper tubes was ≈ 8–12 μm based on SEM imaging.

### Hydrophobic coating

The inverse opal coated copper tubes were oxygen plasma treated for 15 min. Immediately after plasma treatment, the tubes were placed inside a desiccator (2204K5, McMaster-Carr) along with few drops of silane (trichloro(1H,1H,2H,2H-perfluorooctyl)silane, Sigma Aldrich) in a polystyrene petri dish (Falcon™ 351008, Thermo Fisher Scientific). The pressure inside the desiccator was lowered by connecting it to a vacuum/suction line. Once low pressure was established inside the container, silane started to evaporate at which point the vacuum line was disconnected. Intermittently, the vacuum line was reconnected to allow all the liquid silane to evaporate. The evaporated silane in vapor phase was adsorbed on the plasma treated surface and rendered the copper tube hydrophobic.

### Oil impregnation (SLIPS)

The inverse opal coated and hydrophobized copper tube was impregnated with a chemically matched silicone oil (100 cSt at 25 °C, Sigma Aldrich). After impregnation, the tube was oriented vertically upwards for > 12 h to drain the excess lubrication oil via gravity. The lubrication oil that infiltrated the pores was immobilized by capillary forces. The lubricated tube was inspected visually for the absence of excess oil prior to experiment.

## Supplementary Information

Supplementary Information.

## Data Availability

All data generated or analyzed during this study are included in this published article and/or the Supplementary material.
