# Supplementary material for: Enhanced condensation heat transfer using porous silica inverse opal coatings on copper tubes
Source: Sci Rep. 2021 May 21;11:10675. doi: 10.1038/s41598-021-90015-x (PMC8140112; doi:10.1038/s41598-021-90015-x)
Supplement: Supplementary file 1 — Supplementary Information. [file 41598_2021_90015_MOESM1_ESM.pdf]

Supporting Material for

Enhanced condensation heat transfer using porous silica inverse opal coatings on copper tubes

Solomon Adera[1,2,3,*], Lauren Naworski[1], Alana Davitt[1], Nikolaj K. Mandsberg[1,5], Anna V. Shneidman[1], Jack Alvarenga[1], Joanna Aizenberg[1,2,4,*]

[1]*John A. Paulson School of Engineering and Applied Sciences, Harvard University, Cambridge, Massachusetts 02138, USA.*
[2]*Wyss Institute for Biologically Inspired Engineering, Harvard University, Cambridge, Massachusetts 02138, USA.*
[3]*Department of Mechanical Engineering, University of Michigan, Ann Arbor, Michigan 48109, USA.*
[4]*Department of Chemistry and Chemical Biology, Harvard University, Cambridge, Massachusetts 02138, USA.*
[5]*Department of Health Technology,Technical University of Denmark, 2800 Kongens Lyngby, Denmark.*

Address correspondence to sadera@umich.edu and jaiz@seas.harvard.edu

S1. Surface characterization

A copper plate was coated with silica inverse opals using the same colloidal co-assembly technique used for the copper tubes.[1-3] The apparent contact angle of a millimeter-size water droplet was measured using a digital shape analyzer (DSA100, KRÜSS GmbH). Before silane treatment, water spreads instantaneously with vanishing (near zero) apparent contact angle (θ_{app}) as shown in Fig. S1a. When the inverse opal coated copper plate was silanized (hydrophobized *via* silane treatment), the water droplet remained contained with advancing/receding contact angle of 155°/142° (≈13° contact angle hysteresis) (Fig. S1b). When the silanized porous inverse opals were impregnated with silicone oil (100 cSt at 25 °C), the droplet became hemispherical with ≈90° contact angle (Fig. S1c). The advancing/receding contact angle of a millimeter-size water droplet on the nanostructured lubricated surface was 95°/94° (≈1° contact angle hysteresis.

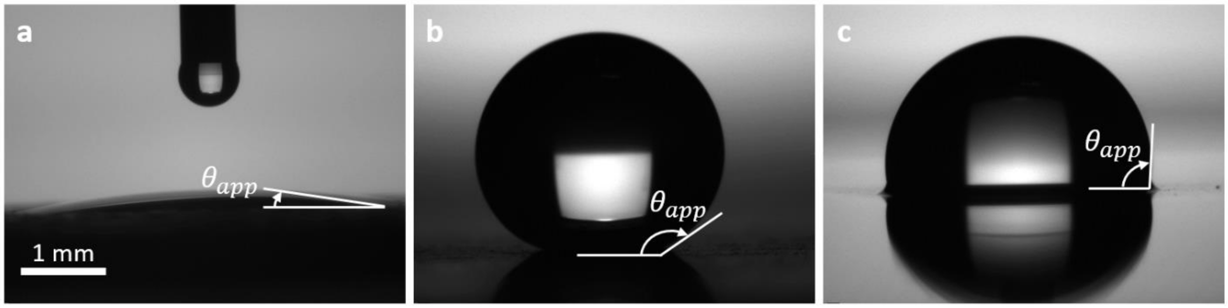

Figure S1. Apparent contact angle. Contact angle of a millimeter size water droplet on a copper surface coated with porous silica inverse opals (a) before silanization, (b) after silanization, and (c) after oil impregnation. Due to the inherent hydrophilicity of silica, water spreads instantaneously with near zero degree contact angle. When the surface was hydrophobized with silane, the advancing/receding contact angle became 155°/142°. When the hydrophobized porous structure was impregnated with a lubrication film, the advancing/receding contact angle became 95°/94° with ≈1° contact angle hysteresis.

More than 15 condensation experiments, each lasting 3-4 h, were conducted on the inverse opal coated copper tubes. No heat transfer performance degradation was observed in our experiments. Additionally, we used SEM to investigate the surface coating (topography) post-condensation. As shown in Fig. S2, the inverse opal coating and surface morphology was nearly intact after repeated condensation experiments. Visually, we did not observe wear and tear on the silica inverse opal coating.

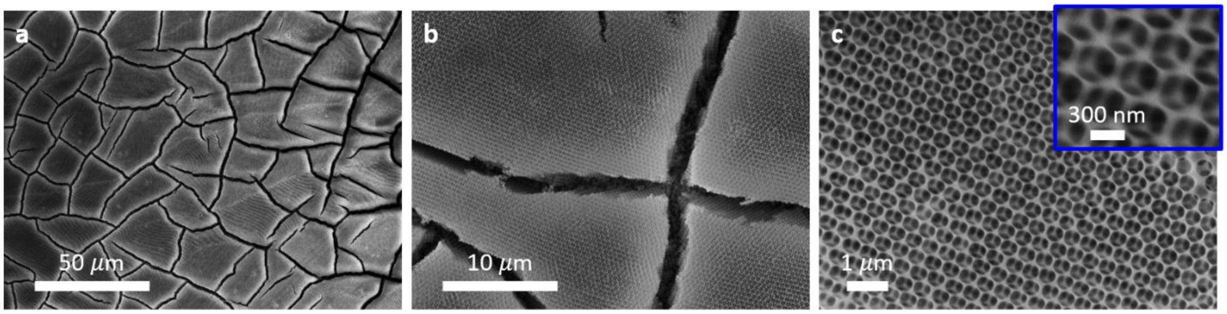

Figure S2. Post-condensation top-down scanning electron microscope (SEM) images of the porous structure (inverse opal) at different magnifications. No visible damage or surface degradation was observed (a-c). The inverse opal coating remained intact on the outer surface of the copper tube and maintained high heat transfer coefficient when the experiments were repeated.

S2. Environmental chamber

A stainless steel cylindrical environmental chamber shown schematically in Fig. S3 was built to experimentally characterize the heat transfer performance of the copper tubes. The environmental chamber was designed to maintain saturated conditions (saturation temperature and pressure) by eliminating NCGs from the system. The nominal diameter and length of the chamber are 15 cm and 45 cm, respectively. The chamber has two flanges (QF100-BK, KJLC) and gasket center rings (QF100−SAVR) to cover the right and left ends of the cylinder using clamps (QF-SDC-AL1). A resistive heating cable with a controller (3641K22, McMaster-Carr) is wrapped around the chamber to heat the walls slightly above saturation temperature to prevent condensation on the internal walls. To reduce heat loss to ambient air, the chamber is insulated externally using fiberglass insulation sheet (9333K71, McMaster-Carr). Calibrated Type K thermocouples (KMQSS-062G-6, Omega Engineering) are fed into the chamber *via* a thermocouple feedthrough (TFT3KY00008A) to measure wet-bulb and dry-bulb temperatures. Additionally, the chamber pressure is measured using a calibrated pressure transducer (275 Mini-Convectron®, MKS instruments).

Hot steam is supplied to the environmental chamber from a custom-made stainless steel vapor generator. A rope heater with a controller is wrapped around the stainless steel vapor generator (diameter ≈ 10 cm, length ≈ 30 cm). The vapor generator along with the rope heater are insulated externally using fiberglass insulation to minimize heat loss to the surrounding air. The vapor supply line is heated using a separate rope heater to prevent steam condensation inside the flexible tubing (321-4-×-24, Swagelok) before reaching the chamber. A stainless steel needle valve (SS-4BG, Swagelok) is used to control the amount of vapor (hot steam) entering the chamber through a flange (QF16X4SWG).

In preparation for the experiment, the vapor generator was filled with DIW. The water in the vapor generator was degassed *via* boiling. The valve connecting the vapor generator and chamber was closed and the rope heater was turned on to boil the water vigorously. After boiling the water for 1-2 min, the pressurized vapor was allowed to escape by opening the valve. Following the pressure release, the valve was closed and the water was boiled for an additional 1-2 min before letting the vapor out again by opening the valve. This procedure was repeated at least 4-5 times before closing the valve and allowing the vapor/liquid mixture inside the vapor generator to reach saturation condition (≈85 °C and ≈59 kPa) by turning off the power supply to the rope heater. The vapor/liquid saturation temperature inside the vapor generator was measured by inserting a calibrated Type K thermocouple. During the experiment, the temperature of the water/vapor mixture was maintained nearly constant at ≈85 °C by adjusting the power-regulating knob on the rope heater.

Chilled water was fed into the environmental chamber through a liquid feedthrough (LFT8B22SWSW). After attaching the copper tube in the water circulation line using union tee fittings (SS-400-3, Swagelok), the entire system was evacuated using a roughing pump (Edwards RV8, Mass-Vac Inc.). A cold trap

(TLR4XI100QF) filled with liquid nitrogen was used to capture water vapor that would otherwise contaminate the oil in the vacuum pump. Chamber pressure was below ambient (negative absolute pressure) with negligible leak rate (<3 Pa/h). A bellow valve (SA0100MVQF), which connects the environmental chamber with the vacuum pump, remained closed during experiment. A separate bellow valve was used to rush air in and bring the chamber to atmospheric pressure after each experimental run which lasted for 3-4 hr. During experiment, the condensate that accumulated at the bottom surface was drained through a drain valve that is located at the bottom of the chamber. After each experimental run, the internal walls of the chamber were dried using Kimwipes (Kimberly-Clark Professional™ 34155, Thermo Fisher Scientific) in preparation for the next experiment.

The environmental chamber has a 4 cm diameter sapphire viewing port (QF40-150-VP). A fiber optic illuminator (SCHOTT-KL1500-LCD, Leica Inc.) is used as a cold light source and DSLR camera (EOS 60D, Canon Inc.) is used to capture images of droplet nucleation, growth, and departure. All tubes except the copper tube were insulated to avoid condensation. The copper tube was maintained below the saturation temperature by circulating chilled water from a water bath heat exchanger (Haskris R050 Recirculating Chiller, Haskris). The flow rate of the cooling water was measured using a factory-calibrated liquid flow meter (10 LPM MAX, Alicat Scientific). Additionally, the temperature of the incoming and outgoing cooling water was measured using calibrated Type J thermocouples (JMQSS-062G-6, Omega Engineering). The data from the thermocouples, pressure transducer, and flow meter were recorded using a data acquisition system (cDAQ-9174, CompactDAQ, National Instruments) that was interfaced with LabVIEW (National Instruments). Steady-state condition was reached when the fluctuation in temperature (wet-bulb, dry-bulb, inlet, and outlet temperatures) and chamber pressure were ≤0.25 °C/min and ≤0.1 kPa/min, respectively. Steady-state data was collected for ≈3-5 min and time averaged for this study.

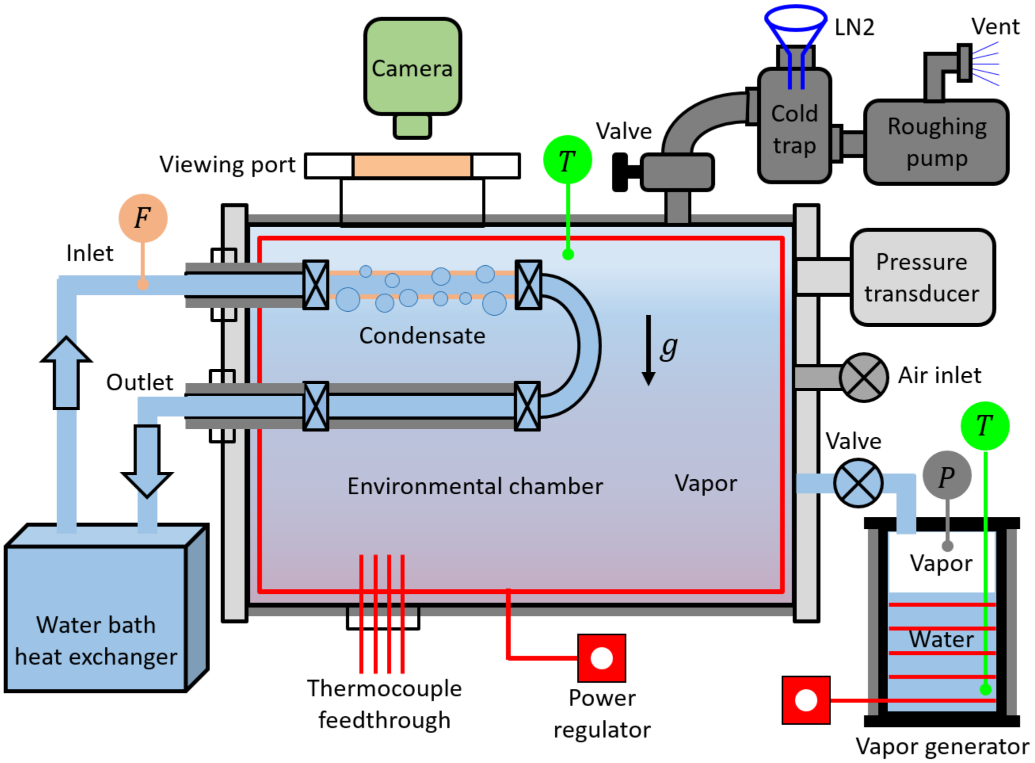

Figure S3. Schematic experimental setup. The chamber is designed to maintain saturation condition inside the chamber. The copper tube was cooled below the saturation temperature by circulating chilled water through the tube. Pure vapor (without noncondensable gases) was supplied to the chamber from a vapor generator to initiate condensation on the copper tube. The environmental chamber was insulated to minimize heat loss to ambient air.

Depending on the surface functionalization, we observed different modes of condensation (Fig. S4). When the smooth copper tube was plasma treated, steam condensed in filmwise mode (Fig. S4a). When the smooth copper tube was hydrophobized, vapor condensed in dropwise mode (Fig. S4b). When the copper tube was coated with silica inverse opals, the condensate seeped into the porous structure. Unlike filmwise condensation, the condensate was transported preferentially in the axial direction towards the ends of the tube through the cracks which present relatively low resistance to fluid flow (Fig, S4c). Arrows in Fig. S4c indicate the preferential axial condensate transport direction. When the copper tube was impregnated with silicone oil, steam condensed by forming discrete water droplets that depart early at smaller radius and higher frequency (Fig. S4d).

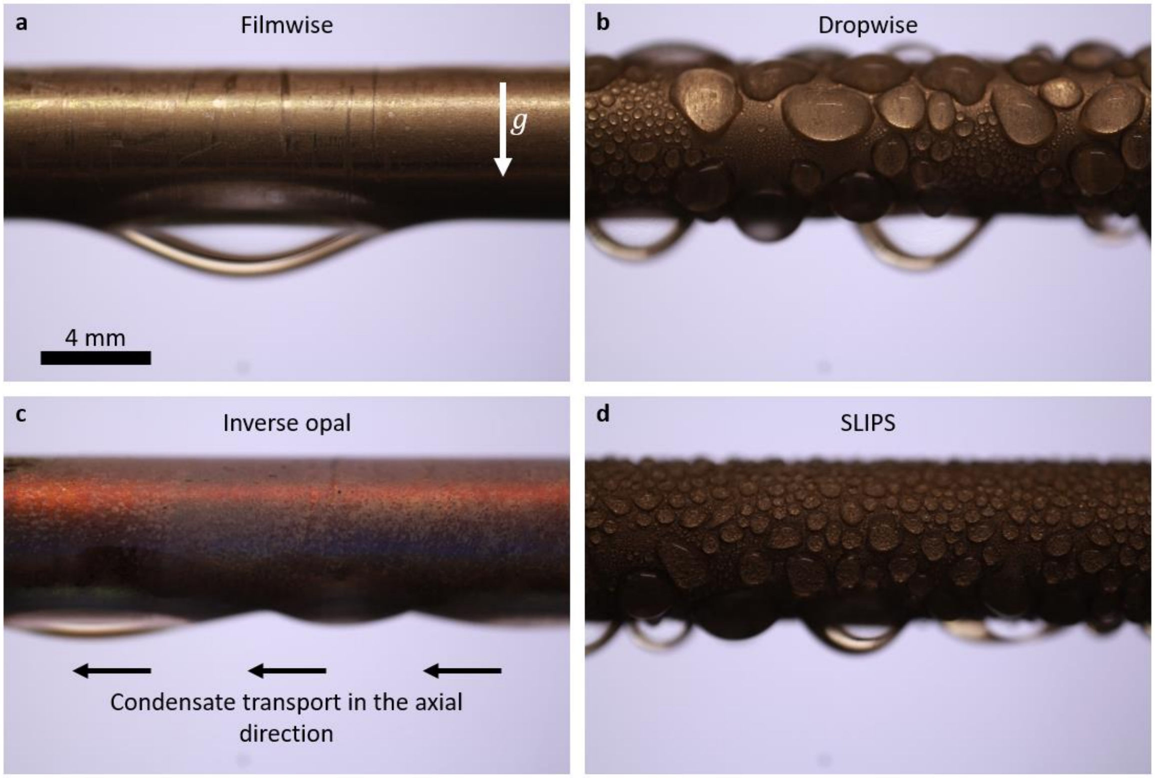

Figure S4. Modes of condensation. Photographic images of (a) filmwise condensation (FWC) on smooth plasma treated (hydrophilic), (b) dropwise condensation (DWC) on smooth hydrophobized (silane treated), (c) inverse opal condensation (IOC) on silica inverse opal coated, and (d) SLIPS condensation on oil-impregnated copper tubes. The arrows in (c) show the direction of the axial transport of the condensate through the interconnected cracks.

S3. Thermocouple calibration

All thermocouples were calibrated prior to experiment by inserting them in a constant temperature water bath and correlating the thermocouple reading with the actual temperature. The water bath was maintained at constant temperature by recirculating the working fluid (Lauda ECO RE 420 G, LAUDA-Brinkmann). The refrigerant cooled heat exchanger has a built-in temperature controller with <0.05 °C accuracy. After setting the temperature on the controller, the working fluid was recirculated by connecting the inlet to the outlet. The thermocouples in the water bath were allowed to reach steady-state (<0.1°C/min) before collecting calibration data using a data acquisition system. The reading from the thermocouple (y-axis,) and the actual/true water bath temperature (x-axis) correlate linearly with <1% root-mean-square error (RMSE) as shown in Fig, S5. Error bars (one standard deviation) are small and not visible in Fig, S5. During experiments, the slope (α) and y-intercept (β) of the calibration curve were used as correction factors to deduce the actual/true temperature (T_{act}) from the thermocouple reading (T_{rdg}) by using the equation

$$T_{\text{act}} = \frac{T_{\text{rdg}} - \beta}{\alpha}. \tag{1}$$

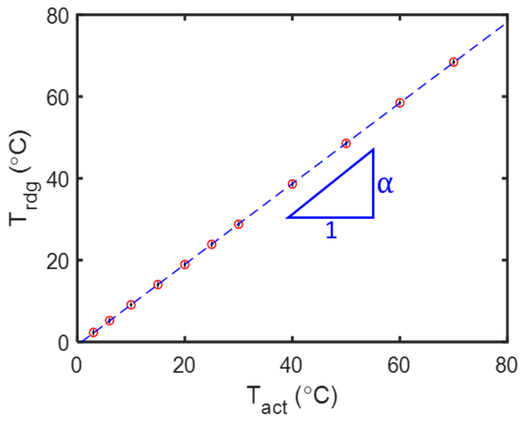

Figure S5. Thermocouple calibration curve. Thermocouples were calibrated prior to experiment by immersing them in a constant temperature water bath. The temperature reading from the thermocouple (T_{rdg}) and the actual/true temperature (T_{act}) correlate linearly with <1% root-mean-square error (RMSE).

S4. Heat transfer model

The wet-bulb temperature was measured by inserting a calibrated thermocouple into a wet cloth that was Kapton taped (7648A726, MaMaster-Carr) at the bottom of the cylindrical chamber while the dry-bulb temperature was measured by hanging a thermocouple probe inside the chamber near the condensing copper tube. The configuration of these two thermocouple probes (wet-bulb and dry-bulb) is shown schematically in Fig. S6. In our experiment, the wet-bulb and dry-bulb temperatures match within the measurement uncertainty (±0.2 °C), indicating saturated condition (absence of NCGs) inside the environmental chamber. The saturation temperature, which was used in subsequent calculations, was deduced by linearly averaging the wet-bulb and dry-bulb temperatures.

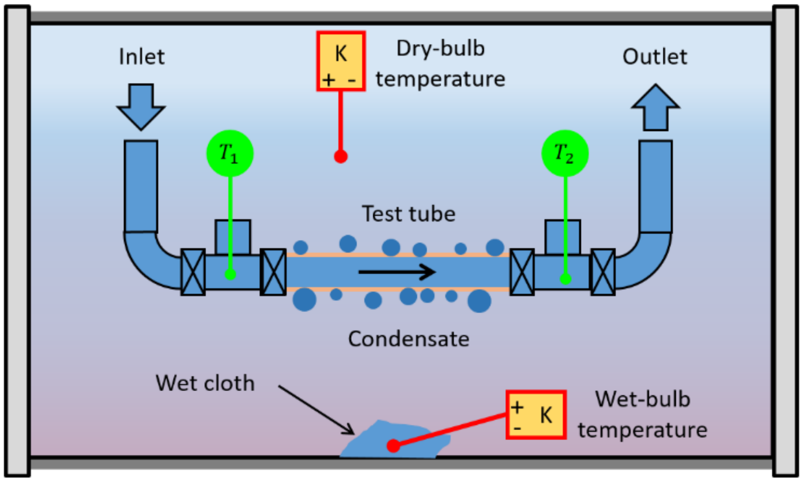

Figure S6. Wet-bulb and dry-bulb temperatures. The wet-bulb temperature was measured by imbedding a thermocouple probe inside a wet cloth while the dry-bulb temperatures was measured by hanging a thermocouple probe near the condensing copper tube inside the environmental chamber. The wet-bulb and dry-bulb temperatures match within ±0.2 °C, indicating saturated conditions inside the chamber.

The temperatures of the incoming (T_1) and outgoing (T_2) cooling water were measured using calibrated Type J thermocouples (JMQSS-062G-6, Omega Engineering). Additionally, the volumetric flow rate was measured using a liquid flow meter. The heat rejected by the vapor as it condenses on the copper tube was

obtained from the sensible heat gained by the circulating chilled water. The heat released per unit area or condensation heat flux (q") is given by

$$q" = \frac{\rho_w \dot{V} c_w (T_2 - T_1)}{\pi d_o L}, \quad (2)$$

where ρ_w, $\dot{V}$, and c_w are density, volumetric flow rate, and specific heat capacity of the cooling water, respectively, d_o is the outer diameter (6.35 mm) and L is the length (60 mm)of the copper tube. Since the phase-change heat transfer in our experiments is neither isothermal nor isoflux, we used logarithmic mean temperature difference (ΔT_{LMTD}) as the average subcooling as

$$\Delta T_{LMTD} = \frac{(T_2 - T_{sat}) - (T_1 - T_{sat})}{\ln\left(\frac{T_2 - T_{sat}}{T_1 - T_{sat}}\right)}, \quad (3)$$

where T_{sat} is the saturation vapor temperature (average of wet-bulb and dry-bulb). Accounting for the forced convection internal flow, conduction through the wall, and phase-change condensation outside the tube, the overall heat transfer coefficient (U_o) is given by

$$U_o = \frac{q"}{\Delta T_{LMTD}}. \quad (4)$$

We estimate the heat transfer coefficient for internal flow by first calculating the diameter-based Reynolds number (Re_D) from the mass flow rate as

$$Re_D = \frac{4\dot{m}_w}{\pi d_i \mu_w}, \quad (5)$$

where d_i is the internal diameter (4.57 mm), μ_w is the viscosity of water, and $\dot{m}_w$ is mass flow rate which can be obtained easily from the measured volume flow rate as

$$\dot{m}_w = \rho_w \dot{V}. \quad (6)$$

To minimize the internal flow (convection) resistance, we used large flow rates (≥5 liters per minute) which resulted in turbulent flow with Re_D>10,000. The friction factor (f) was obtained from the Reynolds number using the Petukhov's correlation for turbulent flow[4] as

$$f = (0.790 \ln(Re_D) - 1.64)^{-2}. \quad (7)$$

The dimensionless diameter-based Nusselt number (Nu_D) was estimated from the friction factor (f) and Reynolds number (Re_D) using the Gnielinski correlation[5] for pipe flow as

$$Nu_D = \frac{(f/8)(Re_D - 1000)Pr}{1 + 12.7(f/8)^{1/2}(Pr^{2/3} - 1)}, \quad (8)$$

where Pr is the dimensionless Prandtl number (ratio of momentum to thermal diffusivity). The above equation (Gnielinski correlation) is accurate within ±10% in the range $0.5 < Pr < 10^6$ and $2300 < Re_D < 5\times10^6$. Furthermore, the Gnielinski correlation can be used in both constant heat flux and constant wall temperature boundary conditions.

The internal heat transfer coefficient (h_i) was calculated from the Nusselt number as

$$h_i = \frac{Nu_D k_w}{d_i}, \quad (9)$$

where k_w is the thermal conductivity of water.

Knowing the values of the convection and the conduction resistances which are depicted schematically in Fig. S7, the steady-state phase-change condensation heat transfer coefficient on the vapor-side (h_c) was calculated as

$$h_c = \left(\frac{1}{U_o} - \frac{A_o}{A_i h_i} - \frac{A_o \ln(d_o/d_i)}{2\pi L k_t}\right)^{-1}, \quad (10)$$

where k_t is the thermal conductivity of the tube, A_i and A_0 are the inner ($\pi d_i L$) and outer ($\pi d_o L$) surface areas of the tube, respectively.

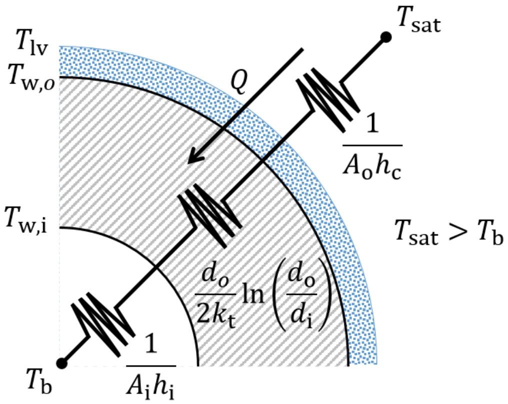

Figure S7. Schematic depiction of the three major thermal resistances, namely the forced convection flow internal thermal resistance ($1/A_\mathrm{i}h_\mathrm{i}$), the conduction resistance through the tube ($d_\mathrm{o}\ln(d_\mathrm{o}/d_\mathrm{i})/2k_\mathrm{t}$), and the vapor-side thermal resistance for phase change heat transfer ($1/A_\mathrm{o}h_\mathrm{c}$). The heat ($Q$) that is released as vapor condenses on the tube is carried away by the forced convection of the bulk liquid.

Rearranging Eq. (10) gives three thermal resistances in series (Fig. S7) as

$$\frac{1}{A_\mathrm{o}U_\mathrm{o}} = \frac{1}{A_\mathrm{i}h_\mathrm{i}} + \frac{\ln(d_\mathrm{o}/d_\mathrm{i})}{2\pi L k_\mathrm{t}} + \frac{1}{A_\mathrm{o}h_\mathrm{c}}, \tag{11}$$

where the left side of the equation represents the overall thermal resistance ($1/A_\mathrm{o}U_\mathrm{o}$). The first ($1/A_\mathrm{i}h_\mathrm{i}$) and last ($1/A_\mathrm{o}h_\mathrm{c}$) terms represent the thermal resistances associated with forced internal flow and phase-change condensation, respectively. The middle term ($\ln(d_\mathrm{o}/d_\mathrm{i})/2\pi L k_\mathrm{t}$) represents the conduction resistance through the walls of the tube in the radial direction. The internal wall temperature of the tube ($T_\mathrm{w,i}$, Fig. S7) can be computed as

$$T_\mathrm{w,i} = T_\mathrm{b} + \frac{Q}{A_\mathrm{i}h_\mathrm{i}}, \tag{12}$$

where Q is the total heat rejected by the vapor ($\dot{m}_\mathrm{w}c_\mathrm{w}(T_2 - T_1)$) and T_b is the bulk temperature of the chilled water which we estimated by averaging the inlet and outlet temperatures of the cooling water as

$$T_\mathrm{b} = (T_1 + T_2)/2. \tag{13}$$

The bulk temperature is the average temperature (averaged over the cross-sectional area) of the internal flow in the pipe. Similarly, the outside wall temperature ($T_\mathrm{w,o}$, Fig. S7) can be estimated from the inside wall temperature as

$$T_\mathrm{w,o} = T_\mathrm{w,i} + \frac{Q\ln(d_\mathrm{o}/d_\mathrm{i})}{2\pi L k_\mathrm{t}}. \tag{14}$$

The temperature difference between the outer wall ($T_\mathrm{w,o}$) and the saturated vapor inside the chamber (T_sat) is the true subcooling which is given by

$$\Delta T = T_\mathrm{sat} - T_\mathrm{w,o}, \tag{15}$$

where ΔT is subcooling. Given that the sensible heat gain in terms of temperature rise of the working fluid is small in our experiments (0.2-2.5 °C temperature difference between inlet and outlet), we evaluated thermophysical properties of the working fluid at the mean/average bulk temperature to simplify the analysis.

S5. Modeling Nusselt laminar film condensation on a pipe

The laminar film condensation on the surface of a single horizontal cylinder of diameter D (Fig. S8a) was first analyzed by Nusselt.[6] The diameter-based average phase-change heat transfer coefficient (averaged over the entire condensing surface of the cylinder) for laminar filmwise condensation ($\bar{h}_\mathrm{c,fwc}$), where the Reynolds number based on the mass flow rate is below 30, is given by the classical Nusselt model[4,6,7] as

$$\overline{h}_{\mathrm{c,fwc}} = 0.729\left(\frac{g\rho_{\mathrm{l}}(\rho_{\mathrm{l}}-\rho_{\mathrm{v}})k_{\mathrm{l}}^{3}h_{\mathrm{fg}}'}{\mu_{\mathrm{l}}D(T_{\mathrm{sat}}-T_{\mathrm{w}})}\right)^{1/4}, \tag{16}$$

where g is the acceleration due to gravity (9.81 m/s^2), h_{fg}' is the modified latent heat of vaporization (enthalpy of phase change), ρ is the density with subscripts l and v for liquid (water in this study) and vapor/steam, respectively, μ_{l} is the dynamic viscosity of the condensate liquid, and T_{sat} and T_{w} are the saturation and wall temperatures, respectively. We used the modified latent heat of vaporization to account for the sensible cooling of the condensate liquid from T_{sat} at the liquid-vapor interface to T_{w} at the pipe outer wall (Fig. S8a-b). For the subcooling effect, we used the Rohsenow[8] correlation, which is given by

$$h_{\mathrm{fg}}' = h_{\mathrm{fg}}(1+0.68\mathrm{Ja}), \tag{17}$$

in which Jakob number (Ja) is a relative measure of the degree of subcooling experienced by the liquid film,

$$\mathrm{Ja} = \frac{c_{\mathrm{p,l}}\left(T_{\mathrm{sat}}-T_{\mathrm{w,o}}\right)}{h_{\mathrm{fg}}}. \tag{18}$$

In the above equation, h_{fg} is the latent heat of vaporization evaluated at the vapor saturation temperature (T_{sat}). We used a reference temperature scheme to account for variable-property effects. Liquid condensate properties (ρ_{l}, μ_{l}, k_{l}) are evaluated at the film temperature (T_{film}), which is the average of the vapor saturation and wall temperatures, that is, $T_{\mathrm{film}} = 1/2(T_{\mathrm{sat}}+T_{\mathrm{w}})$ (Fig. S8b). The ρ_{v} and h_{fg} are evaluated at T_{sat}. Also shown in Fig. S8b is the velocity profile of the condensate liquid film. The velocity profile (quadratic) can be obtained from solving the flow-direction momentum equation for no-slip at the wall and shear free at the liquid-vapor interface. Unlike the velocity profile, the temperature profile, which can be obtained from solving the energy equation, is linear. The boundary conditions for the energy equation (conduction through the condensate film) are the wall temperature at the solid-liquid interface and saturation temperature at the liquid-vapor interface.

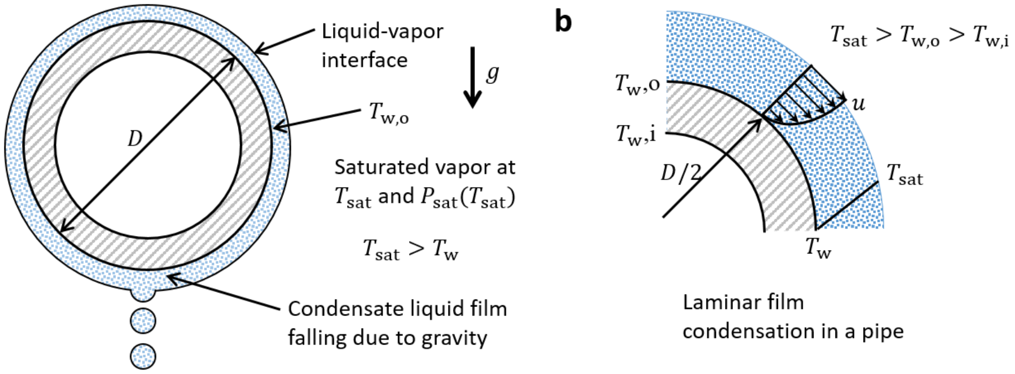

Figure S8. Nusselt laminar film condensation on a cylinder. (a) Condensation commences at the top of the pipe and the condensate film thickness continues to increase from the top to the bottom since condensation continues on the liquid-vapor interface as the condensate travels from the top to the bottom. The surrounding vapor is at a higher temperature (T_{sat}) than the wall temperature (T_{w}). (b) The velocity and temperature profiles of the condensate film. The velocity boundary conditions are the no-slip at the wall and free surface (no shear stress) at the liquid-vapor interface. Nusselt solved the coupled momentum and energy equation in 1916.

S6. Error propagation analysis

The measurement uncertainty (S) on the overall heat transfer coefficient (U_o) is calculated by propagating the instrument uncertainty of each measured quantity. This is given by

$$\frac{S_{U_{\mathrm{o}}}}{U_{\mathrm{o}}} = \sqrt{\left(\frac{S_{\dot{m}_{\mathrm{w}}}}{\dot{m}_{\mathrm{w}}}\right)^2 + \left(\frac{S_{(T_2-T_1)}}{(T_2-T_1)}\right)^2 + \left(\frac{-S_{A_{\mathrm{o}}}}{A_{\mathrm{o}}}\right)^2 + \left(\frac{-S_{\Delta T_{\mathrm{LMTD}}}}{\Delta T_{\mathrm{LMTD}}}\right)^2}. \tag{19}$$

As the phase-change heat transfer coefficient (h_c) is a product of powers, the measurement error is estimated by taking the first partial derivatives of h_c with respect to the overall and internal heat transfer coefficients as

$$\frac{S_{h_c}}{h_c} = \sqrt{\left(\frac{\partial h_c}{\partial U_o}\frac{S_{U_o}}{U_o}\right)^2 + \left(\frac{\partial h_c}{\partial h_i}\frac{S_{h_i}}{h_i}\right)^2}. \quad (20)$$

The first partial derivatives of h_c with respect to the overall heat transfer coefficient (U_o) and internal convection heat transfer coefficient (h_i) are given by

$$\frac{\partial h_c}{\partial U_o} = \frac{{h_i}^2}{(h_i - (A_o/A_i)U_o - R_t A_o U_o h_i)^2}, \quad (21)$$

and

$$\frac{\partial h_c}{\partial h_i} = \frac{-(A_o/A_i){U_o}^2}{(h_i - (A_o/A_i)U_o - R_t A_o U_o h_i)^2}, \quad (22)$$

where R_t is the conduction resistance of the tube in the radial direction. The radial conduction resistance for the cylindrical tube is given by

$$R_t = \frac{\ln(d_o/d_i)}{2\pi k_t}. \quad (23)$$

The error bars reported in this study are obtained by combining system and random errors for one standard deviation. Table S1 summarizes the uncertainty associated with each measured quantity.

Table S1. Uncertainties corresponding to experimental measurement.

**Experimental measurement**	**Uncertainty (±)**
Calibrated thermocouples (T_1, T_2, T_{sat})	<0.2 K
Saturated vapor pressure (P_v)	<2%
Chilled water mass flow rate ($\dot{m}_w$)	<4%
Copper tube surface area (A_o, A_i)	<2%
Gnielinski correlation heat transfer coefficient (h_i)	<10%

References

1 Hatton, B., Mishchenko, L., Davis, S., Sandhage, K. H. & Aizenberg, J. Assembly of large-area, highly ordered, crack-free inverse opal films. *Proc. Natl. Acad. Sci. U. S. A.* **107**, 10354-10359 (2010).

2 Mishchenko, L., Hatton, B., Kolle, M. & Aizenberg, J. Patterning hierarchy in direct and inverse opal crystals. *Small* **8**, 1904-1911 (2012).

3 Phillips, K. R. *et al.* Fabrication of photonic microbricks via crack engineering of colloidal crystals. *Adv. Funct. Mater.* **30**, 1908242-1908211 (2020).

4 Mills, A. F. *Basic Heat and Mass Transfer*. 2nd edn, (Prentice-Hall, 1999).

5 Gnielinski, V. New equations for heat and mass transfer in turbulent pipe and channel flow. *Int. Chem. Eng.* **16**, 359-368 (1976).

6 Nusselt, W. Die Oberflachenkondensation des Wasserdamphes. *Z. Ver. Dtsch. Ing.* **60**, 541-546 (1916).

7 Bejan, A. *Convection heat transfer*. (John wiley & sons, 2013).

8 Rohsenow, W. M. Heat transfer and temperature distribution in laminar film condensation. *Trans. ASME* **78**, 1645-1648 (1956).
